# Cell paintballing using optically targeted coacervate microdroplets†
†Electronic supplementary information (ESI) available. See DOI: 10.1039/c5sc02266e. The datasets corresponding to this manuscript and supporting information are also available at DOI: 10.5523/bris.141i8gcwao5pb1li26zvzdojc3.


**DOI:** 10.1039/c5sc02266e

**Published:** 2015-07-20

**Authors:** James P. K. Armstrong, Sam N. Olof, Monika D. Jakimowicz, Anthony P. Hollander, Stephen Mann, Sean A. Davis, Mervyn J. Miles, Avinash J. Patil, Adam W. Perriman

**Affiliations:** a Bristol Centre for Functional Nanomaterials , University of Bristol , BS8 1FD , UK; b Centre for Organized Matter Chemistry and Centre for Protolife Research , School of Chemistry , University of Bristol , BS8 1TS , UK . Email: avinash.patil@bristol.ac.uk ; Email: chawp@bristol.ac.uk; c School of Cellular and Molecular Medicine , University of Bristol , BS8 1TD , UK; d HH Wills Physics Laboratory , University of Bristol , BS8 1TL , UK

## Abstract

We present a new approach for the directed delivery of biomolecular payloads to individual cells with high spatial precision. This was accomplished *via* active sequestration of proteins, oligonucleotides or molecular dyes into coacervate microdroplets, which were then delivered to specific regions of stem cell membranes using a dynamic holographic assembler, resulting in spontaneous coacervate microdroplet–membrane fusion. The facile preparation, high sequestration efficiency and inherent membrane affinity of the microdroplets make this novel “cell paintballing” technology a highly advantageous option for spatially-directed cell functionalization, with potential applications in single cell stimulation, transfection and differentiation.

## Abstract 2

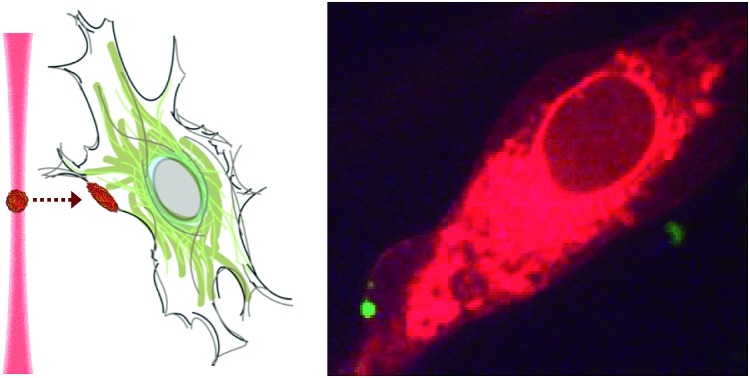
A dynamic holographic assembler was used to “paintball” stem cells with coacervate microdroplets loaded with protein, oligonucleotide or molecular dye.

## Introduction

The emergence of crosscutting techniques such as cellular levitation,^1^ bio-microfluidics^2^ and live-cell microscopy,^3^ as well as advances in single-cell proteomics,^4^ genomics^5^ and spectroscopy,^6^ has created a demand for new approaches to interrogate cells on an individual basis. Moreover, the ability to deliver functional biological payloads to specific regions of individual cells provides an exciting new platform for manipulating single cells, screening drug interactions and studying membrane processes. Current cell functionalization strategies utilize exogenous materials that indiscriminately adhere to, or transverse, the cell membrane. Typically, membrane-active species will possess either a cationic surface charge to facilitate electrostatic interactions with anionic membrane proteoglycans, a hydrophobic anchor to partition into the phospholipid bilayer, or conjugated antibodies to promote specific membrane binding. These membrane functionalization approaches have been used to visualize sub-cellular structure,^7^ provide nutrients during tissue engineering,^8^ improve *in vivo* cell targeting,^9^ facilitate the external manipulation of cells,^10^ and initiate signalling cascades for cancer therapies.^11^ Such biotechnologies are limited to some extent by their indiscriminate nature, with widespread membrane labelling of the entire cell population resulting from bulk phase delivery.

The development of optical tweezer technology, which uses focused laser assemblies to generate optical traps, has enabled precision micromanipulation of micron-sized objects with well-defined three-dimensional control.^12^ This in turn has created a unique opportunity for the development of new microscale vectors for the targeted delivery of biomolecular species. Complex coacervate microdroplets are ideal candidates, as they form spontaneously *via* the self-assembly of oppositely-charged electrolytes, which include a wide range of nucleotides, peptides and saccharides.^13^–^15^ Moreover, they can be isolated and re-suspended from a bulk phase to produce microdroplets with an array of highly unusual physical properties that are ideally suited for the encapsulation and delivery of biomolecular payloads. Significantly, coacervate microdroplets do not possess a membrane, but instead are stabilised by electrostatic, hydrophobic and polymer entanglement interactions between constituent species in a highly crowded environment.^16^ Moreover, the high internal volume fraction of charged electrolytes facilitates the active and selective sequestration of guest species, a diffusion process that is unimpeded by any interfacial barrier. Indeed, encapsulated species have been used to enhance biosynthetic reactions,^17^ perform selective photocatalysis^18^ and template mineralization^19^ and fatty acid membrane assembly.^20^

**Fig. 1:**
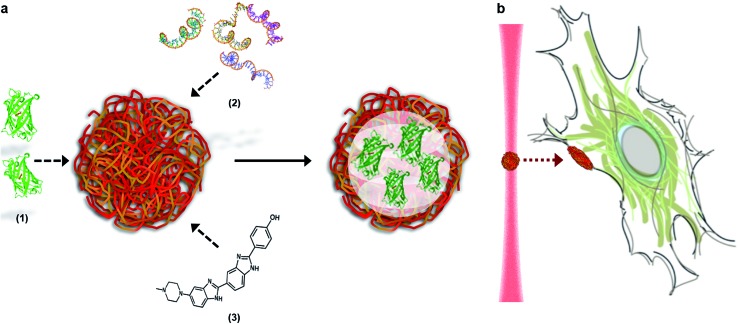
The loading and delivery of coacervate microdroplets. (A) A coacervate phase comprising complexed ATP and PDDA was shown to efficiently sequester proteins (1), oligonucleotides (2) and organic molecules (3) to produce guest-loaded microdroplets. (B) A dynamic holographic assembler was used to optically-trap the microdroplets and deliver the encapsulated payload to targeted areas of the cell membrane.

Here, we describe how the dynamic, non-covalent assembly and membrane-free interface of coacervate microdroplets can be exploited for both active biomolecular cargo loading and direct microdroplet–cell fusion. Furthermore, we demonstrate that the constituent species and encapsulated cargo increase the refractive index of the microdroplets to a level that is compatible with optical tweezing, and show that varying the electrolyte composition allows a high degree of control over microdroplet size and surface charge potential. The results presented herein demonstrate conclusively that coacervate microdroplets can be used as effective vectors for the delivery of proteins, oligonucleotides and small molecules to mesenchymal stem cells, without affecting the viability or multi-lineage differentiation capacity of the functionalised cell. Significantly, we show that a dynamic holographic assembler can be used to “paintball” the cytoplasmic membrane by optically targeting loaded coacervate microdroplets towards selected regions of individual stem cells (Fig. 1).

## Results and discussion

**Fig. 2:**
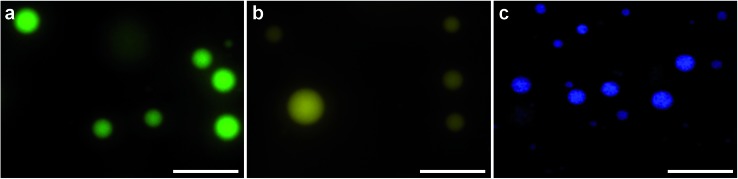
Fluorescence microscopy images of loaded coacervate microdroplets. Fluorescence emission from the encapsulated guest species was observed within microdroplets loaded with (A) eGFP, (B) ssDNA and (C) Hoechst. Scale bars represent 20 μm.

The microdroplets were prepared *via* complex coacervation of anionic adenosine triphosphate (ATP) and cationic poly(diallyldimethylammonium chloride) (PDDA), in a method adapted from Williams *et al.*^21^ (see Experimental details). UV/visible spectroscopy performed on the bulk aqueous solution gave an equilibrium coacervate phase with a 7 : 4 charge ratio of ATP : PDDA (ESI Fig. 1†). As a vector, this coacervate phase was extremely versatile, as demonstrated by the efficient sequestration of biologically-relevant payloads containing significantly different chemical functionalities, structures and molecular weights. Encapsulated species included the cell nucleus staining molecular dye Hoechst 33 258 (Hoechst), fluorescein isothiocyanate (FITC) tagged single-stranded deoxyribonucleic acid (ssDNA) or enhanced green fluorescent protein (eGFP). After sequestration of the guest species, the coacervate bulk phase was isolated and re-suspended in deionized water, which increased the microdroplet monodispersity and stability (ESI Fig. 2†). Statistical image analysis performed on bright field micrographs of each re-suspended coacervate phase found characteristic microdroplet diameters of 2–3 μm (ESI Fig. 3†), while zeta potentiometry measurements gave surface charge potentials of +1.7–10.9 mV, depending on the encapsulated species (Table 1). Fluorescence microscopy images confirmed the presence of eGFP, ssDNA or Hoechst within the coacervate microdroplets (Fig. 2 and ESI Fig. 4†) and UV/visible spectroscopy gave high partition coefficients (*P*) for each of the guest molecules, and showed that coacervate loading was concentration dependent (Table 1 and ESI Fig. 5†). This provided the opportunity to produce coacervate microdroplets with a predefined quantity of the guest species by varying the concentration of the initial loading solution. For instance, at the highest loading levels tested, a 2 μm diameter microdroplet contained approximately 1 × 10^6^ eGFP monomers, 1 × 10^5^ strands of ssDNA, or 2 × 10^6^ molecules of Hoechst.

**Table 1:** Zeta potential measurements and guest molecule loading of ATP : PDDA coacervate microdroplets

	Mean zeta potential/mV	Partition coefficient (*P*)	Number of guest species in a 2 μm diameter microdroplet, at highest concentration tested
Unloaded	9.6 ± 0.5	—	—
ssDNA	3.3 ± 0.5	47	1 × 10^5^
eGFP	1.7 ± 0.5	88	1 × 10^6^
Hoechst	4.6 ± 0.2	583	2 × 10^6^

**Fig. 3:**
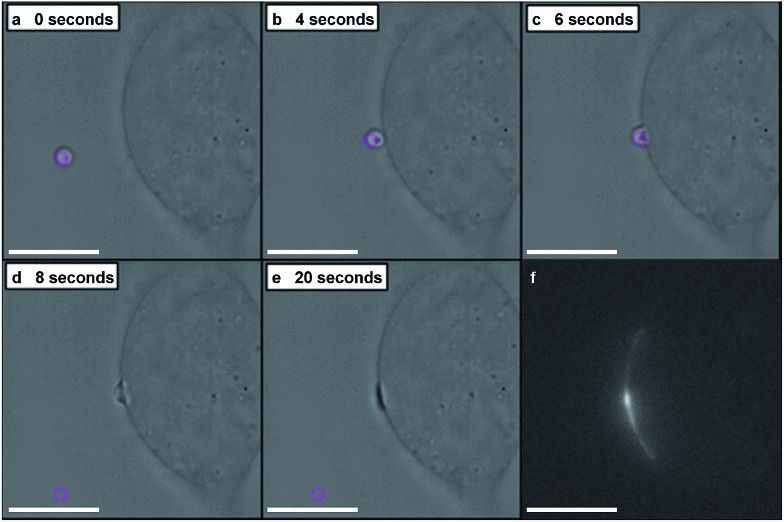
Optically targeted delivery of coacervate microdroplets. (A–E) Bright field microscopy images captured over 20 seconds show an optical trap (purple circle) used to manoeuvre an eGFP-loaded coacervate microdroplet towards an adherent human mesenchymal stem cell (hMSC) resulting in microdroplet–membrane fusion. (F) *In situ* fluorescence microscopy confirmed that the optically-induced fusion event delivered the fluorescent protein payload to the cytoplasmic membrane. Scale bars are 30 μm.

A dynamic holographic assembler, adapted from Gibson *et al.*,^22^ was used to optically trap and manoeuvre coacervate microdroplets in three-dimensions with a maximum velocity of 0.25 mm s^–1^ at an accuracy of 50 nm (ESI Fig. 6†). Beam damage was assayed using *in situ* fluorescence microscopy of a trapped eGFP-loaded microdroplet, which showed negligible loss of fluorescence intensity over 25 minutes (ESI Fig. 7†). Significantly, the dynamic holographic assembler was used to “paintball” human mesenchymal stem cells (hMSCs) by accelerating trapped microdroplets towards the surface of adherent cells, which resulted in spontaneous fusion between the microdroplet and the cell cytoplasmic membrane (Fig. 3A–E and ESI Movies 1 and 2†). Moreover, the dynamic holographic assembler was used to simultaneously deliver multiple microdroplets using independently controlled multiplexed trapping (ESI Movie 3†). Successful fusion events were achieved with both unloaded and loaded microdroplets, which indicated that the initial stage of the microdroplet–membrane interaction was independent of the encapsulated species and mediated by the constituents in the coacervate phase. We postulate that attractive electrostatic interactions between the cationic PDDA molecules at the microdroplet interface and the anionic membrane proteoglycans act to destabilise the coacervate microdroplets, which then coalesce at the phospholipid bilayer boundary.

**Fig. 4:**
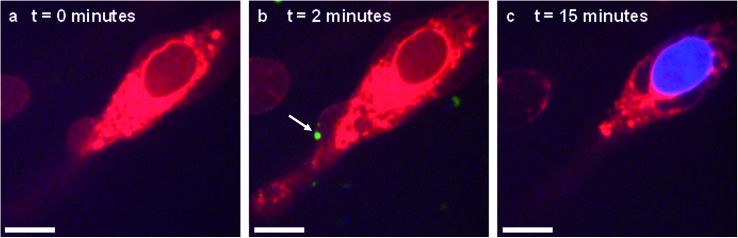
Live-cell confocal microscopy of coacervate microdroplet–membrane interactions. hMSCs visualised using cytoplasm stain (red) were paintballed using coacervate microdroplets doped with fluorescently-tagged ATP (green) and loaded with Hoechst (blue). These time-lapse images show a hMSC (A) prior to addition of microdroplets, (B) immediately after microdroplet–membrane fusion (denoted with white arrow) and (C) with a stained nucleus, indicating gradual internalisation of the Hoechst payload. Scale bars represent 10 μm.

*In situ* fluorescence microscopy was used to observe the guest species at the cell membrane after targeted delivery, which confirmed that optical trapping could be used to deliver functional payloads (Fig. 3F). Live-cell confocal microscopy was performed after spontaneous microdroplet–membrane fusion to assess the delivery and fate of the encapsulated species. Discrete patches of eGFP and ssDNA were observed at the cytoplasmic membrane of paintballed cells, which demonstrated successful delivery of biomolecular payloads (ESI Fig. 8†). Furthermore, microdroplets doped with fluorescent, trinitrophenyl (TNP) tagged ATP showed that the coacervate phase also remained at the cell membrane after paintballing (Fig. 4A and B). The doped coacervate phase, as well as the encapsulated ssDNA or eGFP, persisted at the membrane as discrete patches with minimal lateral diffusion, whereas, microdroplet-delivered Hoechst was gradually internalized and stained the cell nucleus of the paintballed cell within 15 minutes of microdroplet–membrane fusion (Fig. 4C and ESI Fig. 9†). The nuclear staining by Hoechst was an important observation, as it demonstrated that cell paintballing could also be used to deliver functional species beyond the cell membrane. Taken together, these results suggest that the fate of the delivered payload was highly dependent on the chemical and physical properties of the guest species. For instance, Hoechst is a small molecular species with high membrane permeability,^23^ whereas larger biomolecules, such as eGFP, necessitate active endocytic pathways for cellular internalisation.^24^ Importantly, as eGFP and ssDNA do not associate with cells through bulk solution methods, coacervate microdroplet encapsulation offers a new route for the delivery of biomolecules without native membrane affinity.

The effect of paintballing on cell fate was assessed by measuring the viability and multi-lineage differentiation of treated hMSCs. Significantly, Alamar Blue viability assays found no specific cytotoxicity after incubation with up to 100 microdroplets per hMSC, or after the inclusion of the guest species eGFP, ssDNA and Hoechst (ESI Fig. 10†). Post-incubation, the hMSCs continued to proliferate and, importantly, were able to generate differentiated progeny. This was demonstrated through monolayer differentiation experiments, where functionalised hMSCs produced osteoblasts, with visible calcium phosphate deposits, and adipocytes, possessing lipid vacuoles (ESI Fig. 11†). This is a significant result, as it demonstrates that the microdroplet–membrane interactions do not affect the therapeutic utility of the functionalised stem cells.

## Conclusions

In summary, we have demonstrated that a dynamic holographic assembler can be used to optically-manipulate ATP : PDDA coacervate microdroplets, loaded with functional biomolecules, to selected areas of the membrane of individual cells. This “cell paintballing” technology was performed with well-defined payloads of functional guest species, and significantly, coacervate delivery did not affect the viability or multi-lineage differentiation potential of human mesenchymal stem cells. The ability to precisely target bioactive species to selected areas on the membrane of single cells will undoubtedly benefit a wide range of disciplines, with possible applications including the directional stimulation of neurons, the study of lateral diffusion of membrane active drugs, the measurement of membrane receptor–ligand forces and the selective transfection or differentiation of individual cells.

## Experimental details

### Materials

All reagents were purchased from Sigma Aldrich, UK, unless stated otherwise. ssDNA was purchased from Eurofins Genomics and possessed the nucleotide sequence GTTAGCAGCCGGATCTCAGTGGT with a 3′-fluorescein isothiocyanate tag. eGFP was expressed in house, using BL21 competent *Escherichia coli*, transformed with the plasmid vector pET45b(+) (Novagen, Germany). The bacteria were cultured in LB medium with carbenicillin (Apollo Scientific, UK) and expression was induced using isopropylthiogalactosidase (Apollo Scientific, Japan). A Polytron PT2500 homogenizer (Kinematica, Germany) was used to lyse the bacteria in a pH 8 lysis buffer containing 50 mM NaH_2_PO_4_, 300 mM NaCl, 10 mM imidazole and 200 μM phenylmethanesulphonyl fluoride. The lysate was purified using nickel nitriloacetic acid (Qiagen, UK) then dialysed into deionized water using 12–14 kDa molecular weight cut-off dialysis tubing (Medicell International, UK).

### Preparation and characterisation of loaded microdroplets

Coacervation was induced by mixing 200 μL of 25 mM ATP, 200 μL of 25 mM PDDA and 200 μL of either eGFP, ssDNA, Hoechst or deionized water (for unloaded coacervates). All stocks were prepared in deionized water at pH 8.0, and the PDDA concentration was based upon the mass of the repeat unit. The resulting turbid suspension was centrifuged at 4300 g for five minutes to isolate a pellet of coacervate and payload, which comprised approximately 0.5% of the initial reagent volume. Microdroplets were formed by re-suspending the pellet in 400 μL of fresh deionized water at pH 8.0. Doped microdroplets were prepared by using 7.5% (v/v) TNP–ATP in place of ATP in the initial coacervation reaction.

The electrophoretic mobility of the microdroplets was measured in 10 mM HEPES at pH 7.3 using a ZetaSizer Nano ZS (Malvern Instruments, UK). To calculate the microdroplet zeta potential, these data were fitted to Henry's equation with the Smoluchowski approximation and values of 0.8872 cP and 1.33 for the viscosity and dielectric constant, respectively. Depletion assays were performed upon the post-coacervation supernatant to calculate the quantity of unbound ATP and the mass concentration of unsequestered guest species (*C*_s_). UV/visible spectroscopy was used to detect ATP (260 nm), eGFP (488 nm), ssDNA (496 nm) and Hoechst (342 nm). The volume of the supernatant (*V*_s_) and the coacervate (*V*_c_) was used to calculate the mass concentration of sequestered guest species (*C*_c_), across a range of concentrations. This dose response was shown to be linear, thus these data sets were used to plot the mass of sequestered guest species as a function of the mass of unsequestered guest species, from which the gradient (*m*) was used to calculate the partition coefficient (*P*) (eqn (1)).

1 *P* = (*C*_c_/*C*_s_) = *m* × (*V*_s_/*V*_c_)

Bright field and fluorescence imaging of loaded microdroplets was performed using a 100× objective lens on a DMI 3000 inverted microscope (Leica, UK). The coacervates were imaged on glass coverslips that had been coated for one hour in a 2% (v/v) 2-[methoxy(polyethyleneoxy)propyl]trimethoxysilane (abcr GmbH, Germany) in toluene. The excitation filter used depended upon the encapsulated guest species: eGFP (450–490 nm), ssDNA (515–560 nm), TNP–ATP (450–490 nm) and Hoechst (340–380 nm). Line profile analysis and particle sizing was performed using ImageJ software (NIH, USA). For the latter, bright field microscopy images were converted to 8 bit and then a threshold was applied to identify microdroplets with a diameter of between 0.5 and 500 μm. The microdroplets were then outlined and compared with the original image, with manual inspection used to eliminate any mistakenly identified microdroplets. 250 microdroplets were analysed for each microdroplet system.

### Mesenchymal stem cell culture and cell paintballing

hMSCs were harvested from the proximal femur bone marrow of osteoarthritic patients undergoing total hip replacement surgery, in full accordance with Bristol Southmead Hospital Research Ethics Committee guidelines (reference #078/01). Cells were cultured in monolayer up to passage five at 37 °C and 5% carbon dioxide. The expansion media used was low glucose Dulbecco's Modified Eagle's Medium (DMEM) containing pyridoxine–HCl and NaHCO_3_ with 100 units mL^–1^ penicillin, 100 μg mL^–1^ streptomycin, 2 mM GlutaMAX supplement (Invitrogen, USA), 10% (v/v) foetal bovine serum and 5 ng mL^–1^ freshly supplemented rhFGF (Peprotech, USA). For the cell paintballing experiments, coacervate microdroplets were introduced to the cells in a minimal medium comprising of 10 mM HEPES buffer and 0.1 mM calcium chloride at pH 8.0. At this point, either (1) targeted delivery with optical tweezers was performed, (2) microdroplet–membrane interactions were imaged using confocal microscopy or (3) the microdroplets were incubated for 15 minutes at room temperature, before washing the cells and returning them to expansion media at 37 °C.

### Optically-targeted fusion

hMSCs were plated onto 5 mm diameter petri dishes with glass substrate (MatTek, USA) and allowed to adhere in expansion media. Cell paintballing was performed by introducing between 0.5–10 μL of loaded coacervate microdroplets to the cells in 1 mL of minimal medium, at the edge of the petri dish. The sample was visualised using a 1.4 numerical aperture, 100× Plan-Neofluar oil immersion objective lens (Zeiss, Germany) on an Axiovert 200 inverted microscope (Zeiss, Germany). Individual coacervate microdroplets were trapped using a diode-pumped Ventus laser (Laser Quantum, UK), with an emission wavelength of 1064 nm and a maximum power output of 3 W. The laser beam was expanded to fill an electrically-addressed X10468-07 spatial light modulator (Hamamatsu, Japan), operating at 203 Hz, with hologram calculations performed in under 1 millisecond. An MS2000 motorized xyz stage (Applied Scientific Instruments, USA) was used to manoeuvre the microdroplets in three-dimensions and induce fusion with individual cells. Bright field microscopy images were captured using a Firewire CMOS camera (Allied Vision, Canada), with videos taken with a frame rate of approximately 400 Hz. Fluorescence microscopy was performed using an X-Cite 120 fluorescent lamp (Excelitas, USA) and a Rolera EM-C2 camera (Q Imaging, Canada). The stage, laser trap and image capture was controlled using a program written in house for LabVIEW software (National Instruments, USA).

### Confocal microscopy of microdroplet–cell interactions

hMSCs were plated onto 5 mm diameter petri dishes with glass substrate (MatTek, USA) and allowed to adhere in expansion media. The media was then aspirated and each dish was incubated for 5 minutes with 1 mL staining media comprising of low glucose DMEM containing pyridoxine–HCl and NaHCO_3_ with 100 units mL^–1^ penicillin, 100 μg mL^–1^ streptomycin, 2 mM GlutaMAX supplement and 0.5 μM Cell Tracker Deep Red CMPTPX Dye (Life Technologies, USA). Cell paintballing was performed by introducing between 0.5–10 μL of loaded coacervate microdroplets to the cells in 1 mL of minimal medium. Spontaneous fusion events between microdroplets and cells were observed using an Ultraview Spinning Disk confocal microscope (Perkin Elmer, UK) attached to a DMI 6000 inverted microscope (Leica, UK). A 40× objective lens was used throughout, with a 488 nm argon laser used for eGFP, ssDNA and TNP–ATP, a 405 nm diode used for Hoechst and a 640 nm diode for cells stained with deep red. Images were captured and processed using Volocity software (Perkin Elmer, UK).

### Alamar Blue assay

hMSCs were seeded into 96 well plates, with 5000 cells per well for all test and control samples, and 0, 100, 500, 1000, 1500, 2000, 3000 or 4000 cells per well for a standard curve. Cells from three patients were used in this study and each sample, control and standard was run in duplicate. These cells were left to adhere in expansion DMEM media at 37 °C for 24 hours, before cell paintballing was performed using 1, 10 or 100 loaded coacervate microdroplets per cell, in 250 μL of minimal medium, along with a control group of just minimal medium. After the 15 minute incubation, the microdroplets were aspirated and replaced with 100 μL of expansion medium, supplemented with 1% (v/v) Alamar Blue (Life Technologies, USA). The wells were then analysed using a microplate reader by exciting at 580 nm and measuring fluorescence emission at 610 nm. The intensity values were converted to cell survival values using the standard curve, and the specific cytotoxicity of each coacervate microdroplet system was determined by normalizing the data to the minimal media control.

### Multi-lineage differentiation assay

hMSCs from one patient were seeded into 24 well plates, with 74 000 cells per well for osteogenesis and 370 000 cells per well for adipogenesis. The cells were cultured for 24 hours, before half of the wells in each set were incubated with 0.5 μL of microdroplets in 1 mL of minimal medium for 15 minutes. The cells were returned to expansion medium and cultured for a further 24 hours, before being cultured in either (1) minimal medium comprising of α-MEM containing NaHCO_3_ with 100 units mL^–1^ penicillin, 100 μg mL^–1^ streptomycin, 2 mM GlutaMAX supplement and 10% (v/v) foetal bovine serum, (2) minimal medium freshly supplemented with 10 μL mL^–1^ StemXVivo adipogenic supplement (R&D Systems, UK) or (3) minimal medium freshly supplemented with 50 μL mL^–1^ StemXVivo osteogenic supplement (R&D Systems, UK). The media was changed twice a week for three weeks, before each well was washed with 500 μL of phosphate buffered saline (PBS), in preparation for fixing and staining. Each well in the osteogenesis group was incubated with 500 μL of ice-cold 70% ethanol at 4 °C for one hour. A 13.75 mg mL^–1^ aqueous solution of Alizarin Red was stirred overnight, adjusted to pH 4.1 using potassium hydroxide and filtered to remove any aggregates. The ethanol fixative was aspirated and each well was incubated with 500 μL of Alizarin Red solution at room temperature for five minutes and then washed five times with 500 μL aliquots of PBS. Each well in the adipogenesis group was incubated with 500 μL of 4% paraformaldehyde in PBS at room temperature for 30 minutes. The paraformaldehyde was aspirated and each well was washed with 500 μL aliquots of PBS and 60% isopropanol. A 5 mg mL^–1^ solution of Oil Red in 60% (v/v) isopropanol was stirred overnight, diluted to 3 mg mL^–1^ using dH_2_O and filtered to remove any aggregates. Each well was incubated with 400 μL of Oil Red solution at room temperature for 30 minutes and then washed once using 500 μL aliquots of 60% isopropanol. For both groups, bright field images were captured using a 10× objective lens on a DMIRB inverted microscope (Leica, UK).

## Supplementary Material

Supplementary movie

Supplementary movie

Supplementary movie

Supplementary information
