# Supplementary material for: Cell paintballing using optically targeted coacervate microdroplets
Source: Chem Sci. 2015 Jul 20;6(11):6106–11. doi: 10.1039/c5sc02266e (PMC6054073; doi:10.1039/c5sc02266e)
Supplement: Supplementary file 4 [file SC-006-C5SC02266E-s004.pdf]

Electronic Supplementary Material (ESI) for Chemical Science.
This journal is © The Royal Society of Chemistry 2015

Cell paintballing using optically targeted coacervate microdroplets

J. P. K. Armstrong [abc†], S. N. Olof [abd†‡], M. D. Jakimowicz [abcd], A. P. Hollander [c§], S. Mann [b], S. A. Davis [b], M. J. Miles [d], A. J. Patil [b*] and A. W. Perriman [bc*]

a. Bristol Centre for Functional Nanomaterials, University of Bristol, BS8 1FD, UK.
b. Centre for Organized Matter Chemistry and Centre for Protolife Research, School of Chemistry, University of Bristol, BS8 1TS, UK.
c. School of Cellular and Molecular Medicine, University of Bristol, BS8 1TD, UK.
d. HH Wills Physics Laboratory, University of Bristol, BS8 1TL, UK.

Supplementary Data

Supplementary Figure 1. Measuring the quantity of uncomplexed ATP. 2
Supplementary Figure 2. Bright field microscopy of coacervate microdroplets before and after re-suspension suspension. 3
Supplementary Figure 3. Sizing the coacervate microdroplets. 4
Supplementary Figure 4. Analysing the fluorescence intensity of loaded microdroplets. 5
Supplementary Figure 5. Measuring the payload sequestration profile. 6
Supplementary Figure 6. Configuration of the dynamic holographic assembler. 7
Supplementary Figure 7. Monitoring the denaturation of sequestered eGFP. 8
Supplementary Figure 8. Live-cell confocal microscopy of microdroplet-membrane interactions. 9
Supplementary Figure 9. Confocal fluorescence microscopy of Hoechst loaded microdroplet delivery. 10
Supplementary Figure 10. Effect of microdroplet delivery upon cell viability. 11
Supplementary Figure 11. Testing the differentiation capacity of paintballed hMSCs. 12
Supplementary Movie 1. Cell paintballing using loaded coacervate microdroplets. 13
Supplementary Movie 2. Cell paintballing using unloaded coacervate microdroplets. 13
Supplementary Movie 3. Cell paintballing using multiple coacervate microdroplets. 13

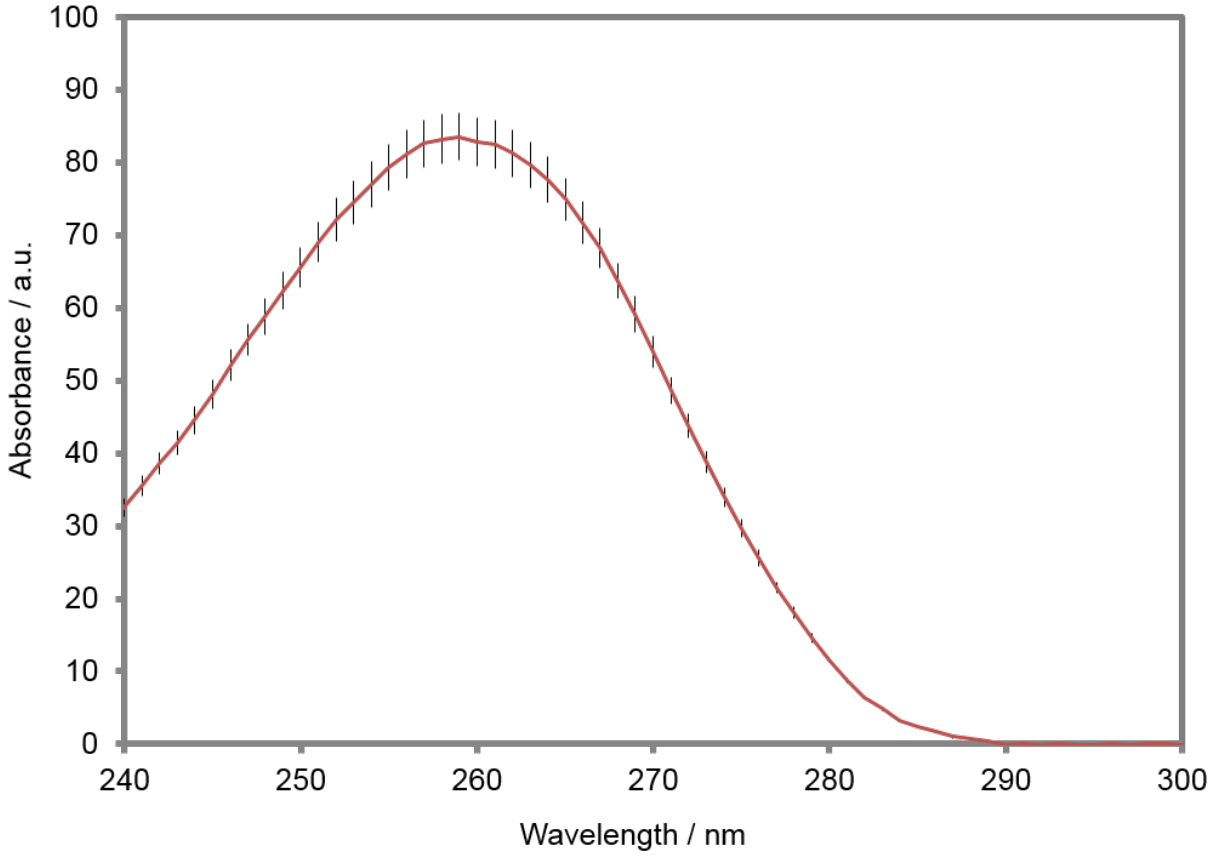

Supplementary Figure 1. Measuring the quantity of uncomplexed ATP. The coacervate phase was synthesised using equivalent volumes of 25 mM ATP and 25 mM PDDA, with the concentration of the latter calculated based upon the mass of the repeat unit. At pH 8, ATP possesses three negative charges while the PDDA repeat unit has one, giving an initial charge stoichiometry of 3 : 1. UV/visible spectroscopy was used to measure the absorbance of the supernatant from centrifuged coacervate microdroplets. In each of the four samples, a peak was detected at 260 nm, which was attributed to uncomplexed ATP (on average, 42% of the initial reagent solution). With the remaining 58% of ATP forming a coacervate with PDDA, the charge ratio was reduced to 7 : 4 (ATP : PDDA).

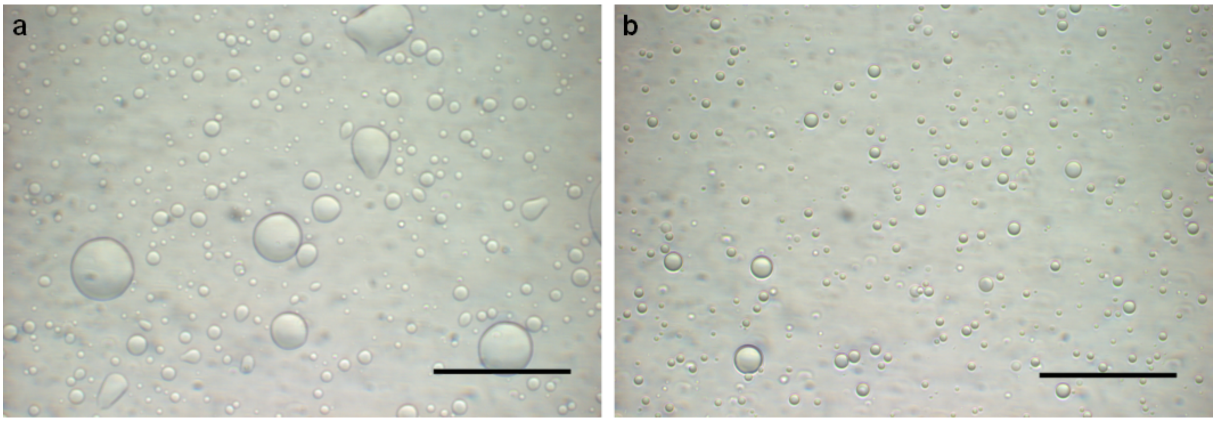

Supplementary Figure 2. Bright field microscopy of coacervate microdroplets before and after re-suspension. (**a**) After coacervation, the ATP:PDDA microdroplets readily wetted the glass surface, (**b**) re-suspension yielded microdroplets that were more stable against coalescence with each other and with the glass substrate. Scale bars represent 100 µm.

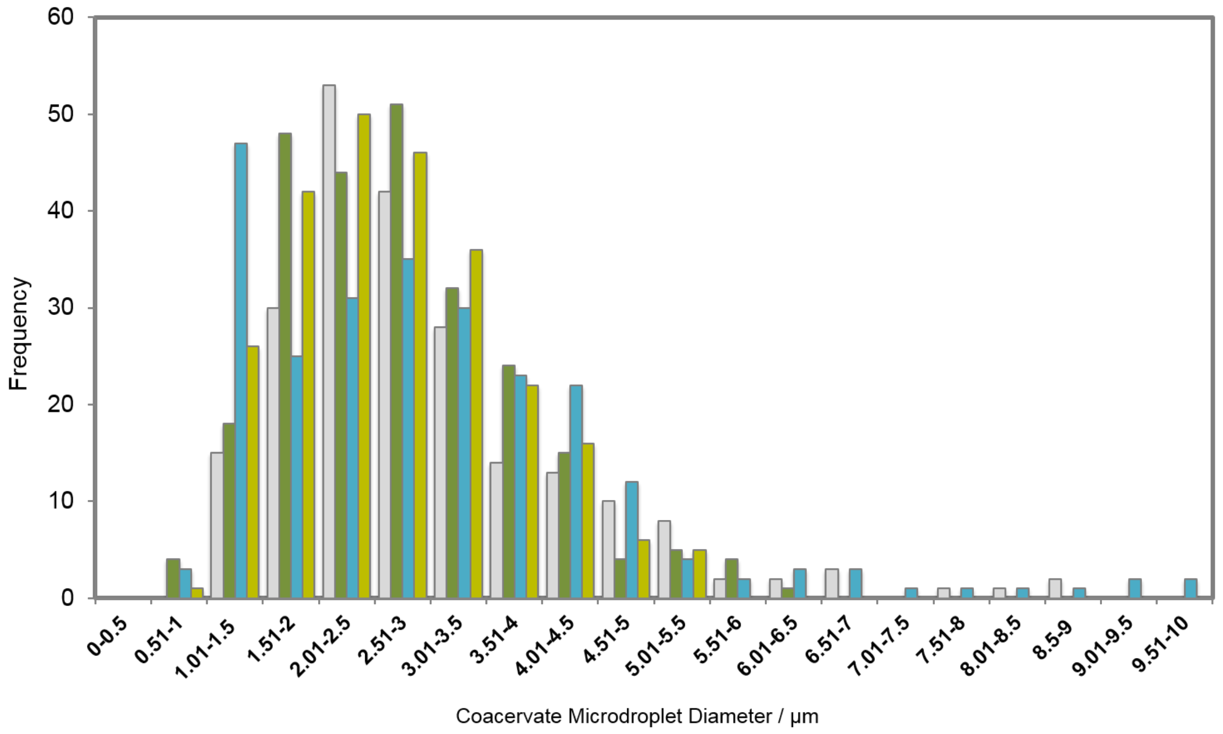

Supplementary Figure 3. Particle size distribution of the coacervate microdroplets. Statistical image analysis was used to measure the coacervate microdroplet size when unloaded (3.0 ± 1.5 µm, grey columns) or sequestered with eGFP (2.7 ± 1.1 µm, green columns), Hoechst (3.1 ± 1.8 µm, blue columns) or ssDNA (2.7 ± 1.0 µm, yellow columns).

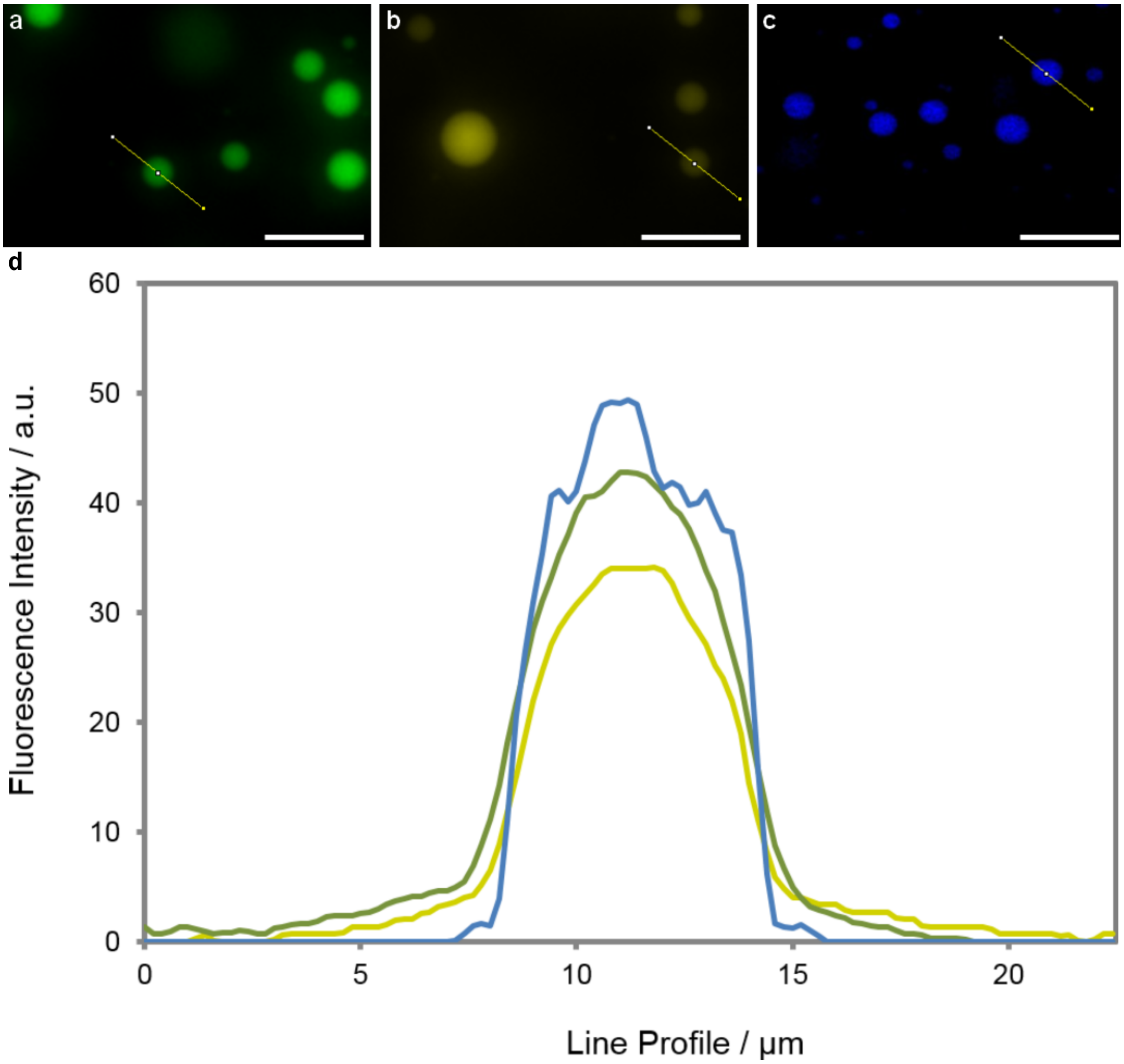

Supplementary Figure 4. Analysing the fluorescence intensity distribution in the loaded microdroplets. Line profile image analysis was performed on fluorescence microscopy images of (**a**) eGFP, (**b**) ssDNA and (**c**) Hoechst. Scale bars represent 200 µm. (**d**) This analysis demonstrated that fluorescence intensity originating from the eGFP (green trace), ssDNA (yellow trace) and Hoechst (blue trace) was located entirely within the coacervate microdroplet phase.

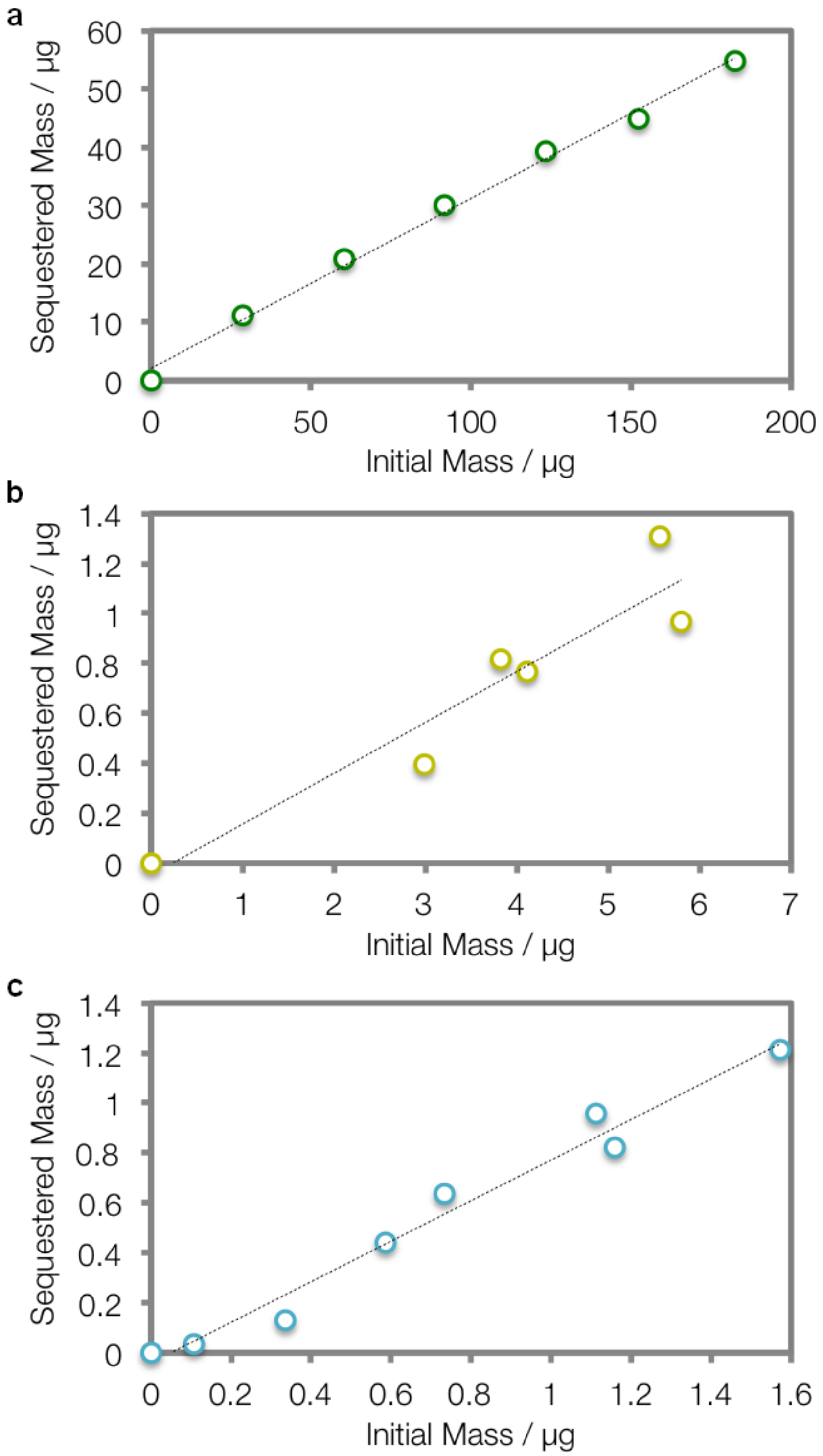

Supplementary Figure 5. Evaluating the payload sequestration profile. The payload mass encapsulated into the coacervate phase was analysed by centrifuging the loaded microdroplets and analysing the supernatant using UV/visible spectroscopy for (**a**) eGFP, (**b**) ssDNA and (**c**) Hoechst. This depletion dosimetry analysis showed a linear response across the range of concentrations tested.

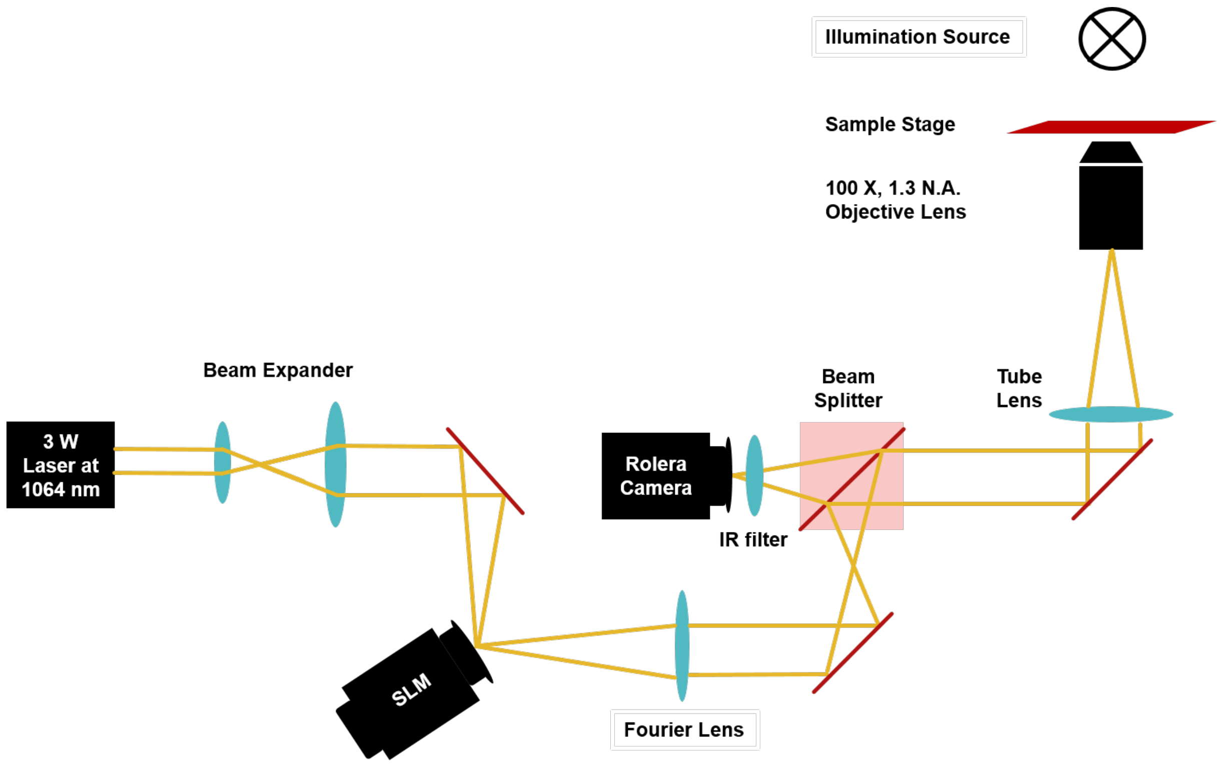

Supplementary Figure 6. Configuration of the dynamic holographic assembler. A Spatial Light Modulator (SLM) coupled with a computer was used to generate real-time phase holograms from an expanded 1064 nm laser beam, which were projected onto the sample stage as optical traps. An illumination source and objective lens were used to project the image from the sample onto a camera. This set up allowed for simultaneous manipulation of the optical trap and video capture.

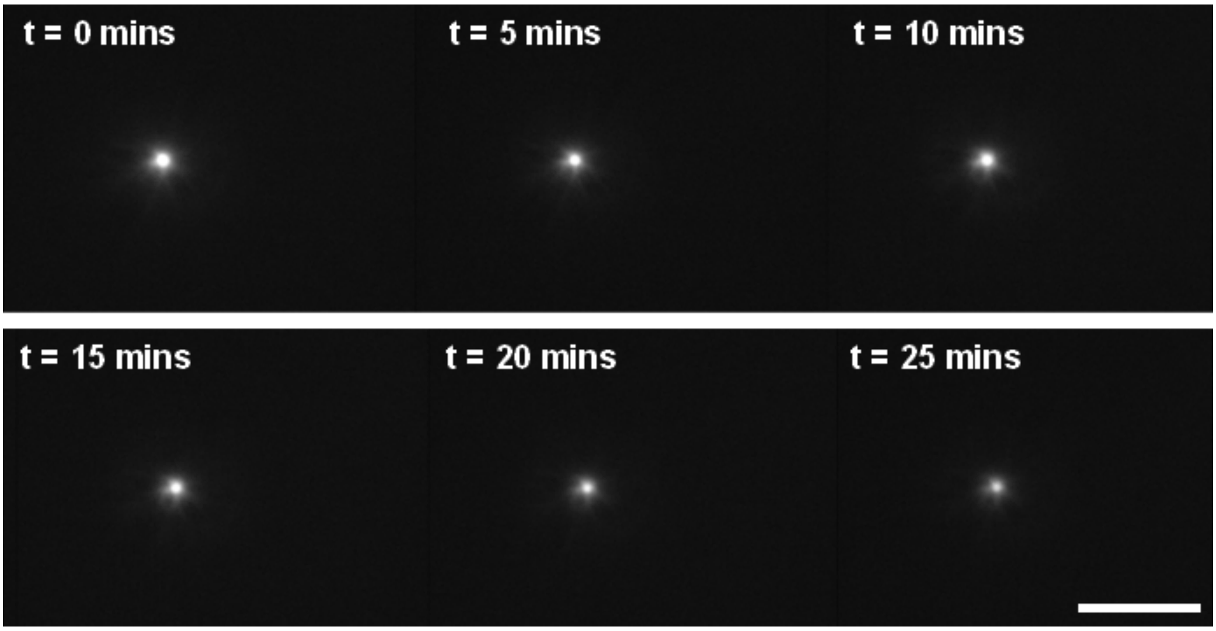

Supplementary Figure 7. eGFP stability in an optical trap. *In situ* fluorescence microscopy was used to monitor the fluorescence of eGFP-loaded coacervate microdroplets, held in an optical trap. Minimal loss in fluorescence intensity was observed over 25 minutes, which indicates that the optical trap had negligible effect upon the structure and function of the encapsulated eGFP. Moreover, this trapping period far exceeded the trapping time required for cell paintballing (< 1 minute). The scale bar represents 30 µm for all images.

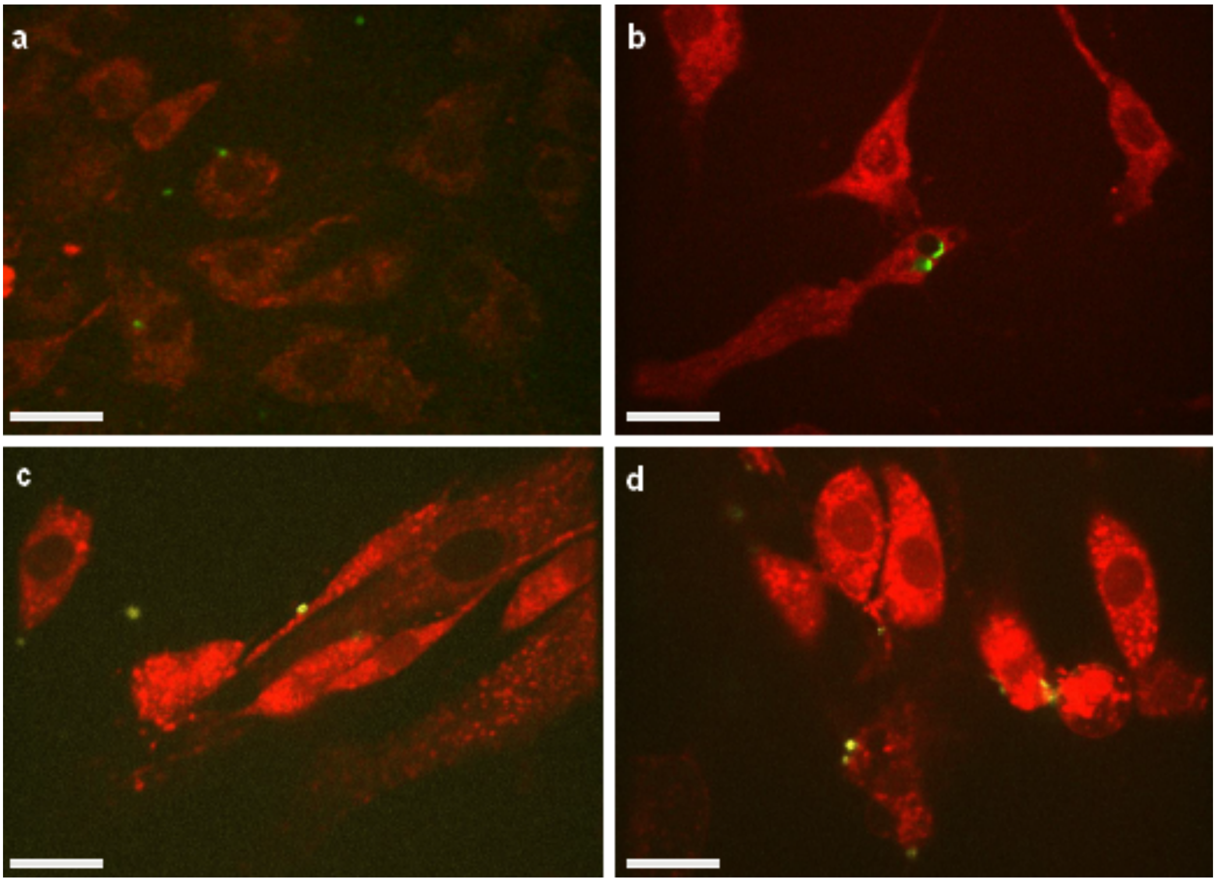

Supplementary Figure 8. Live-cell confocal microscopy of microdroplet-membrane interactions. hMSCs visualised using deep red cytoplasm stain were paintballed using coacervate microdroplets loaded with (**a-b**) eGFP (green) or (**c-d**) ssDNA (yellow). In each case, spontaneous interactions were observed between the coacervate microdroplets and the cell cytoplasmic membrane. Scale bars represent 50 μm.

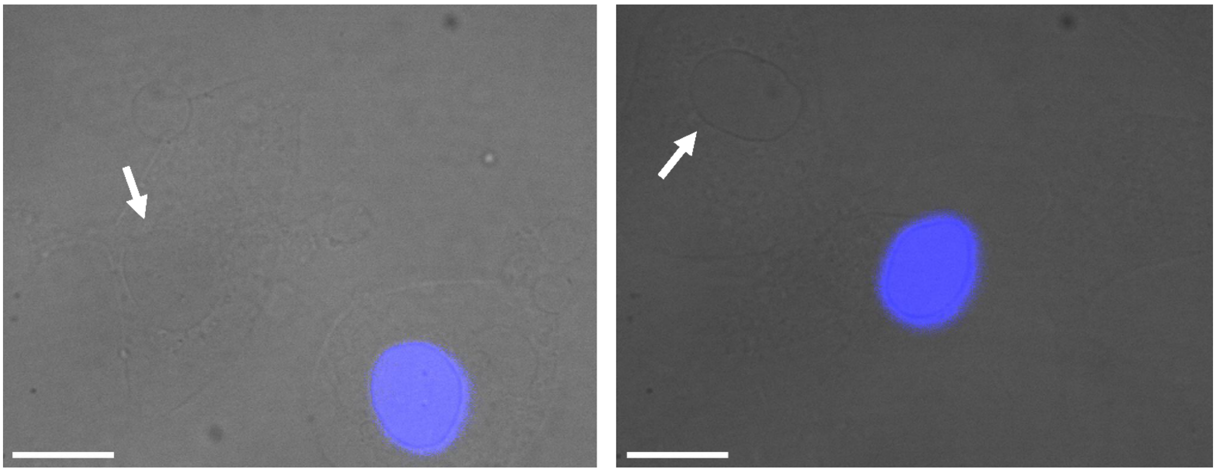

Supplementary Figure 9. Confocal fluorescence microscopy of Hoechst-loaded microdroplet delivery. Non-targeted delivery of Hoechst-loaded coacervate microdroplets resulted in staining of nuclei of certain hMSCs (blue), while others remained unstained (denoted by white arrow). This was evidence for coacervate-mediated delivery resulting from microdroplet-membrane fusion, rather than diffusion from the bulk media. Scale bars represent 170 µm.

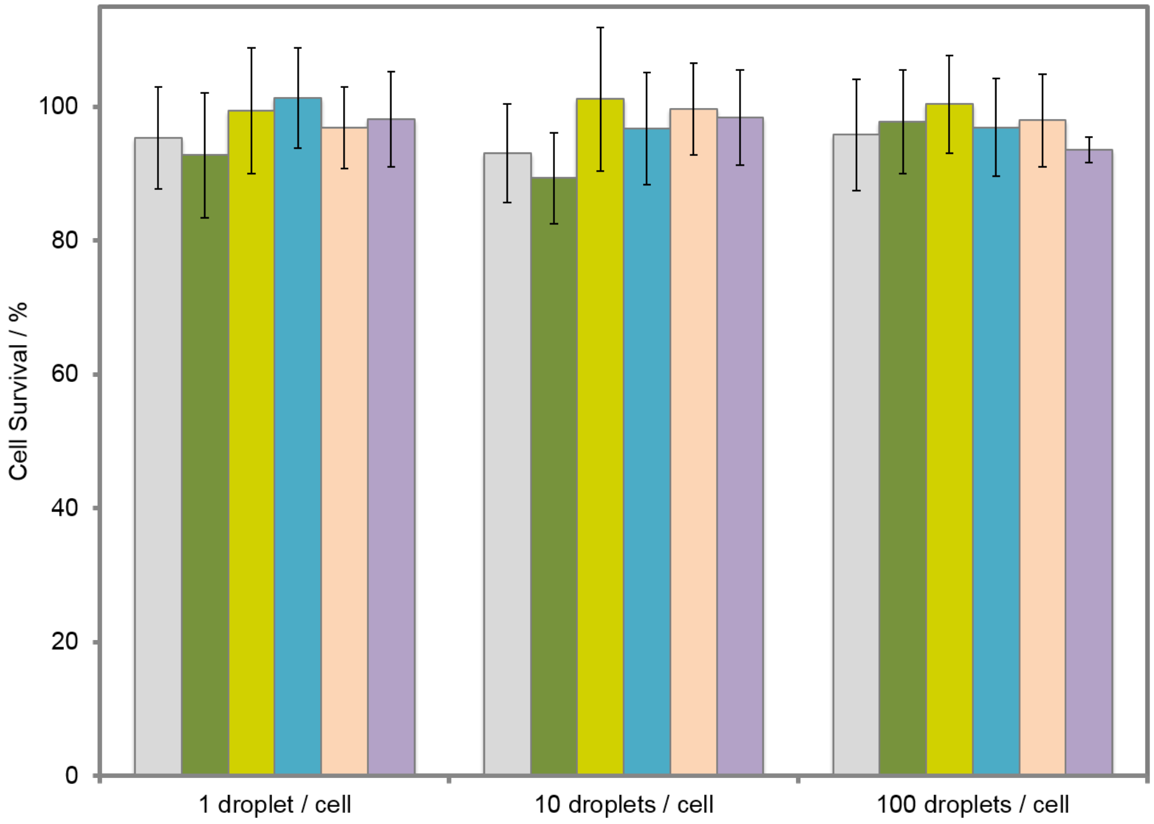

Supplementary Figure 10. Effects of microdroplet delivery upon cell viability. An Alamar Blue viability assay was performed after a 15 minute incubation at a microdroplets to cell ratio of 1:1, 10:1 and 100:1. Under these conditions, the unloaded microdroplets (grey column) and microdroplets loaded with eGFP (green column), ssDNA (yellow column) or Hoechst (blue column) exhibited similar survival rates to a 15 minute incubation in just minimal media. Using the same assay, the corresponding quantities of ATP (orange column) and PDDA (purple column) were also found to have no specific cytotoxicity.

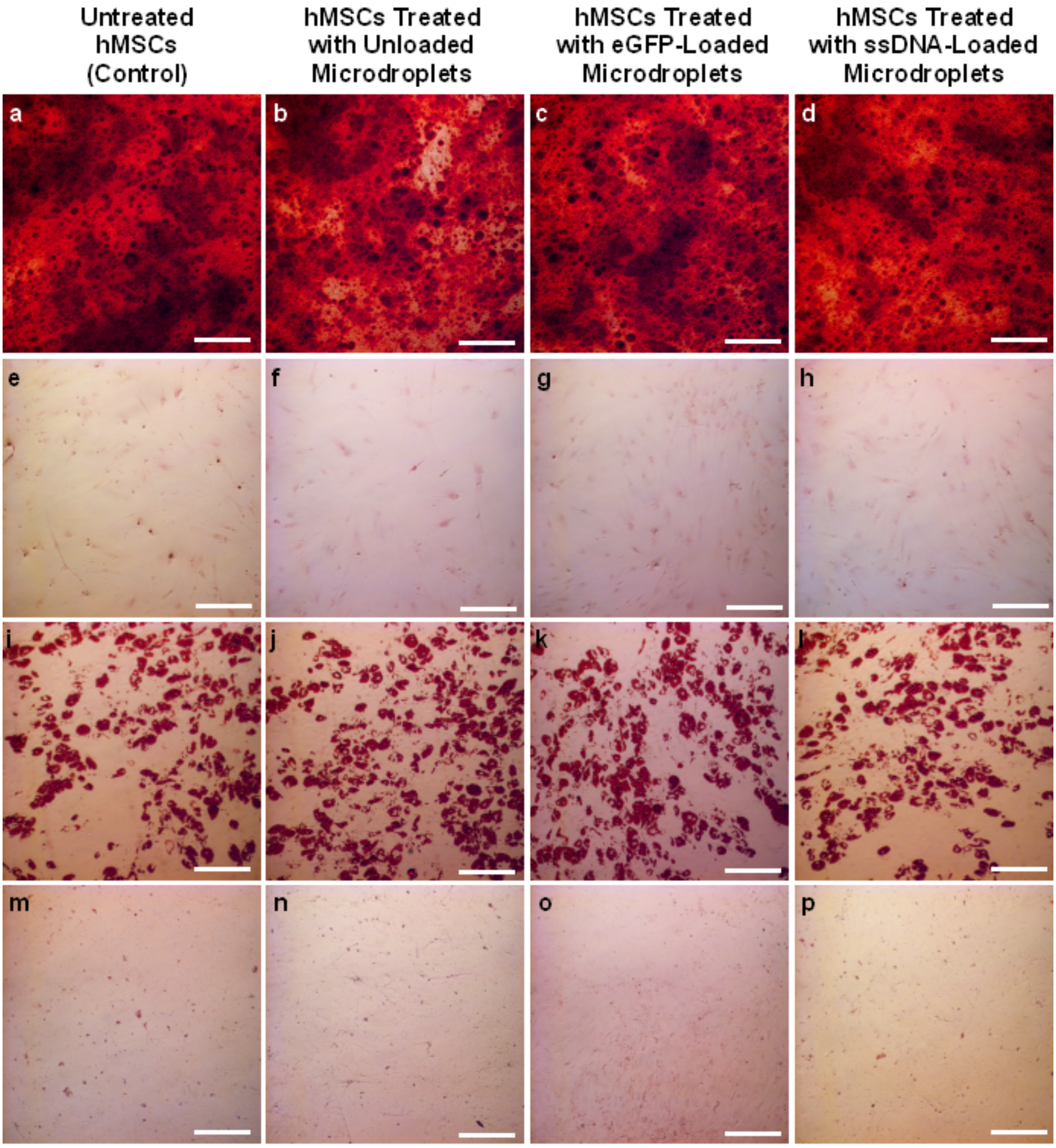

Supplementary Figure 11. Testing the differentiation capacity of paintballed hMSCs. The multi-lineage differentiation capacity of hMSCs paintballed for 15 minutes was compared with untreated hMSCs. Osteogenesis was tested by supplementing the media with (**a-d**) osteogenic factors or (**e-h**) with no additives, similarly, adipogenesis was tested by supplementing the media with (**i-l**) adipogenic growth factors or (**m-p**) with no additives. Alazarin Red was used to stain calcium deposited by osteoblasts, while Oil Red was used to visualise fatty vacuoles present in the adipocytes. The scale bar in all images represent 100 µm.

Supplementary Movie Captions

Supplementary Movie 1. Cell paintballing using loaded coacervate microdroplets. Coacervate microdroplets loaded with eGFP were optically trapped and accelerated towards an adherent hMSC to induce sequential microdroplet-membrane fusion events.

Supplementary Movie 2. Cell paintballing using unloaded coacervate microdroplets. Coacervate microdroplets comprised of only ATP and PDDA used to induce microdroplet-membrane fusion, which is evidence that the cellular interaction is not reliant upon the sequestered payload.

Supplementary Movie 3. Cell paintballing using multiple coacervate microdroplets. Three coacervate microdroplets loaded with Hoechst were simultaneously trapped and delivered to the cell membrane in tandem.
